# Computerized QT and QTc Measurements From Bedside ICU Monitors Are Similar to Those Derived From a Standard 12‐Lead ECG

**DOI:** 10.1111/anec.70031

**Published:** 2024-12-08

**Authors:** Arthur Murray, Karolina Ho, Thomas J. Hoffmann, Gopika K. Ganesh, Shelvin Prasad, Sarah Berger, Cass Sandoval, Amy Larsen, Hildy Schell‐Chaple, Michele M. Pelter

**Affiliations:** ^1^ Benioff Children's Hospital‐Oakland Oakland California USA; ^2^ UCSF Health University of California San Francisco California USA; ^3^ Epidemiology and Biostatistics, School of Medicine and Office of Research, School of Nursing University of California San Francisco San Francisco California USA; ^4^ Kaiser Permanente San Rafael California USA; ^5^ Adult Critical Care, UCSF Health University of California San Francisco California USA; ^6^ Center for Nursing Excellence & Innovation, UCSF Health University of California San Francisco San Francisco California USA; ^7^ ECG Monitoring Research Lab, Department of Physiological Nursing, School of Nursing University of California San Francisco San Francisco California USA

## Abstract

QT/QTc prolongation is associated with an increased risk for torsade de pointes. In at‐risk hospitalized patients, it is common to obtain a standard 12‐lead electrocardiogram (ECG) for this assessment, but this interrupts patient care. Our hospital recently introduced bedside monitors in the intensive care unit (ICU) with continuous QT/QTc software. However, only four of the seven available ECG leads are used.

**Purpose:**

Evaluate the agreement between computerized QT/QTc measurements from the bedside monitor (four leads) and a time‐matched standard 12‐lead ECG.

**Design:**

Prospective observational study in three adult ICUs.

**Methods:**

QT/QTc measurements were obtained from a convenience sample, and the two ECG types were ≤ 30 min apart. Agreement was evaluated using Bland–Altman analysis.

**Results:**

A total of 120 patients were evaluated for inclusion, and 60 (50%) had a 12‐lead ECG for comparison. The mean bias difference for QT measurements was not statistically different (*β* = −2.47, 95% CI = 5.50 to −11.05; *p* = 0.44; limits of agreement (LOA) = −64.37 to 59.44). Similar non‐statistical differences were observed for QTc (*β* = −3.20, 95% CI = 5.50 to −11.05; *p* = 0.44; LOA = −67.43 to 61.03).

**Conclusion:**

There was good agreement for both QT and QTc measurements between the two methods. These pilot data are promising and suggest QT/QTc measurements from bedside monitors (four leads) may be an acceptable alternative to obtaining additional standard 12‐lead ECGs. Given that half of the ICU patients screened did not have a 12‐lead ECG recorded, bedside monitor QT/QTc's could identify at‐risk patients. However, an evaluation in a larger sample and non‐ICU patients is warranted.

## Graphical abstract

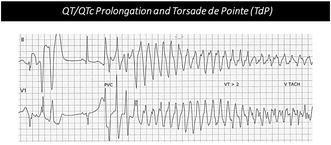

## 1 Introduction

Torsade de pointes (TdP) is a polymorphic ventricular tachycardia that can lead to syncope and even sudden death. The term “TdP” refers to the characteristic electrocardiographic (ECG) pattern that translated, means twisting of the points, which refers to the characteristic QRS complex pattern seen with this arrhythmia. TdP was first identified in 1966 (Dessertenne 1966) and was found to be linked to QT interval prolongation. The entire QT interval represents both the depolarization and repolarization phase of the cardiac cycle. QT/QTc prolongation causes premature action potentials in the ventricular myocytes during the late phase of depolarization, which increases the risk for TdP (Drew et al. 2010). QT/QTc prolongation can appear in a primary or secondary form. The most common primary type is congenital, so called long‐QT syndrome, which can be seen among families, but can also occur in the general population. The secondary type is an “acquired” form, typically associated with medications that prolong the QT/QTc and can lead to the same untoward outcomes as the primary form (i.e., deterioration to ventricular fibrillation and even sudden death) (Drew et al. 2010; Sandau et al. 2017).

In hospitalized patients, the acquired form of QT/QTc prolongation is of particular concern. For example, among intensive care unit (ICU) patients, the acquired type of QT/QTc is prolonged because of clinical and pharmacological factors that are common among critically ill patients (Table 1). One study found a 24% prevalence rate (QT > 500 ms > 15 min), in 1039 consecutive ICU patients (Pickham et al. 2012). In this study, patients with QT/QTc prolongation had a longer length of stay (276 vs. 132 h, *p* < 0.0005) and higher risk of mortality (OR: 2.99; 95% CI 1.1–8.1) as compared to patients without QT prolongation. Others have reported that there is considerable QTc variability in ICU patient during an admission (Janssen, Rijkenberg, and van der Voort 2016) and one study showed a diurnal variation (Dobson et al. 2009). The later presumably because of the timing of routine medication administration during hospitalization (e.g., AM and PM), which can change the QT/QTc.

**TABLE 1:** Risk factors for torsade de pointes in hospitalized patients (Dessertenne 1966).

Clinical factors
QTc > 500 ms
Use of drugs known to prolong the QT interval
Heart disease (heart failure, myocardial; infarction)
Advanced age
Electrolyte disturbance
Hypokalemia
Hypomagnesemia
Hypocalcemia
Treatment with diuretics
Hepatic dysfunction
Bradycardia (sinus, heart block, incomplete heart block with pauses)
Premature ventricular complexes especially short‐long‐short cycles
Congenital long QT syndrome

The most recently published Practice Standards for In‐hospital ECG Monitoring identified QT/QTc interval monitoring as a high priority in at‐risk patients and recommended that hospitals establish uniform protocols for QT/QTc monitoring (Sandau et al. 2017). Specific recommendations include defining a standard procedure for serial measurements; defining criteria for ECG lead(s) selection: consistent use of the same lead(s) for subsequent measurements; educating clinical staff on how to identify the onset of the QRS and the end of the T‐wave; documentation every 8–12 h and more frequently when QT/QTc prolonging drug(s) are administered; and/or when known patient risk factors are present (Sandau et al. 2017). While there are known demographic and clinical characteristics that place patients “at‐risk”, it is routine nursing practice to measure the QT/QTc in all ICU patients with bedside ECG monitoring. This measurement is typically performed by nurses, typically in the one or two ECG leads displayed on the bedside monitor at the start of each shift either by hand, or if available electronic calipers. In addition, physicians and/or providers may also request that measurements be obtained from a standard 12‐lead ECG(s) when there is heightened concerned in select patients. The rational for using a standard 12‐lead ECG is that all 12 ECG leads are used and the QT/QTc is calculated automatically by a software algorithm. However, the steps necessary to obtain a standard 12‐lead ECG can disrupt the flow of patient care, delay findings, increase costs, and a standard 12‐lead ECG is only a 10‐s “snapshot” assessment; thus, there are limitations to this approach.

Given the incidence and dynamic nature of QT/QTc prolongation among ICU patients, continuous measurements would be ideal. Recently, software that continuously and automatically measures the QT/QTc, has been introduced in some bedside ICU monitors (Helfenbein et al. 2006, 2007). One ICU‐based study found a significant correlation between automatically derived QTc values from a standard 12‐lead ECG and QTc values derived from the bedside monitor (Janssen, Rijkenberg, and van der Voort 2016). We are aware of only this one prior ICU‐based study; therefore, validation in a new ICU cohort would be of value. In addition, because the bedside monitor software at our hospital calculates the QT/QTc in only four (I, II, III, and VI) of the seven available ECG leads (i.e., augmented unipolar limb leads are not used), a comparison of bedside monitor derived QT/QTc's to a standard 12‐lead ECG would also be important. Therefore, the purpose of this study was to evaluate the agreement between computerized QT/QTc measurements from the bedside monitor (four leads) and a standard 12‐lead ECG obtained ≤ 30 min apart.

## 2 Methods

### 2.1 Study Design

This was a prospective observational study conducted at a 600‐bed academic medical center. Three adult ICU types were included: cardiac, medical/surgical, and neurological (medical/surgical). The Institutional Review Board approved the study (IRB# 21–34,690) with a waiver of patient consent due to the purely observational nature of the study, and we did not collect private health information from patients or nurse‐related variables.

### 2.2 Sample

The QT/QTc data were obtained in adult (> 18 years) ICU patients from one of three ICU types: (1) cardiac (*n* = 28 beds); medical/surgical (*n* = 32 beds); and neurological (*n* = 29 beds). We collected patient age, sex, and ICU unit type to characterize the sample. The unit of analysis for this study was computerized QT/QTc measurements generated from bedside monitors (four‐lead) and standard 12‐lead ECGs. The latter were obtained by hospital personnel as part of routine patient care.

### 2.3 Data Collection

Our research team collected data on five different days over a 4‐week period. We attempted to collect QT/QTc data from all patients admitted to a unit on the day of data collection. Computerized QT/QTc measurement comparisons were made between a standard 12‐lead ECG obtained from a hospital device as part of routine clinical care and the ICU bedside monitor (details below). If a patient did not have a standard 12‐lead ECG available, they were excluded from the study. We identified the QT/QTc from a hospital acquired standard 12‐lead ECG located in the patient's chart and then used the date and time to compare the QT/QTc measurement derived from the bedside monitor. Measurements between the two methods were compared within 30 min of each other to ensure that medications, and/or clinical factors that could change the QT/QTc did not influence measurements.

### 2.4 Computerized QT/QTc Measurements

#### 2.4.1 Bedside Monitor QT/QTc Measurements

The bedside ECG monitor had QT/QTc software installed (IntelleVue MX800; Philips Healthcare, Cambridge, MA). The QT/QTc values were displayed on the bedside monitor and automatically saved at one‐minute time intervals. While the bedside monitor records seven ECG leads (i.e., I, II, III, aVR, aVL, aVF, and a V lead [default VI]), the software calculates QT/QTc only in leads I, II, III, and the V lead, generating a “global” QT/QTc (Helfenbein et al. 2006, 2007). Every 15‐s, the algorithm performs a QT analysis to determine the average heart rate in order to calculate the QTc using Bazett formula (QTc = QT interval in seconds/ÖRR interval in seconds). When the QT/QTc cannot be reliably analyzed by the software (i.e., atrial fibrillation, flat T‐waves, artifact, small R‐waves, or QT out of range [< 200 or > 800 ms]), an inoperative message alert occurs, and neither the QT, nor QTc is calculated.

#### 2.4.2 Standard 12‐Lead QT/QTc Measurements

At the start of the study, the standard 12‐lead ECG cart used in the hospital was the MAC 5500 Resting ECG Analysis System (GE Medical Systems Technologies; GE Healthcare, Milwaukee, WI). In this study, we will refer to this standard 12‐lead ECG device as device #1. In the second half of the study, the hospital purchased new standard 12‐lead ECG devices (Philips DXL; Philips Healthcare, Andover, MA). In this study, we will refer to this standard 12‐lead ECG device as device #2. Of note, device #2 is the same manufacturer as the bedside ICU monitor. Of note, while the standard 12‐lead ECG device changed during the study, the same bedside monitor was used during the entire study period.

Table 2 shows a summary and comparison of how the algorithm for each manufacturer's device calculates the QT/QTc interval. As with the bedside monitor, all the devices used the Bazett's formula to calculate the QT/QTc.

**TABLE 2:** Comparison of how the algorithm of each electrocardiographic device measures QT/QTc.

Device manufacturer	QT/QTc measurement method
Device #1	Uses all 12‐leads ventricular rate is computed by counting the number of beats detected and dividing by the time difference between the first and last beats QT interval is measured from the earliest detection of depolarization in any lead to the latest detection of repolarization in any lead. Bazett's formula
Device #2	Uses all 12 ECG leads median QT value in “reliable leads.” A lead is considered reliable if the beat‐by‐beat onset/offset determinations have a low variance. This helps to eliminate leads with small amplitudes and high respiratory variation, as well as leads with high noise content Algorithm locates the nadir of the intersection of T and U QT is measured individually and then combined into a global measurement Calculates both Bazett and Fridericia; hospital uses Bazett's
Intensive Care Unit Bedside ECG Monitor (same manufacturer as Device #2); used during the entire study period	Uses leads I, II, III, and the V lead (VI at our hospital) All QRS complexes detected by the beat detection algorithm within a discrete 15‐s time period are saved for subsequent QT interval analysis. In order to calculate the QTc interval, an averaged heart rate (QT‐HR) is generated. QT‐HR is computed using all the valid beats in the 15‐s window used for the QT interval measurement. QTc is then calculated using the rate correction formula selected. The QTc interval is also measured in milliseconds

### 2.5 Comparisons Between Bedside Monitor and Standard 12‐Lead ECG

QT/QTc values are reported in milliseconds (ms). The date and time of the QT/QTc from the standard 12‐lead ECG was used to identify an ECG at the same date/time from the bedside monitor to make comparisons. In a small number of instances, the computerized bedside measurements were not calculated at the exact time of the standard 12‐lead ECG because motion artifact was present in the ECG signal. In these cases, a bedside monitor QT/QTc was obtained as close in time as possible just prior to, or after the standard 12‐lead ECG, which was typically within minutes. An attempt was made to obtain QT/QTc measurements in all the patients admitted to the ICU on the day of data collection.

### 2.6 Statistical Analysis

The characteristics of the sample were analyzed using SPSS (version 29.0.0.0; IBM Corporation, Armonk, NY). R version 4.0.0 (Vienna, Austria) was used for the primary analysis that compared QT/QTc values between the bedside ECG monitor and the standard 12‐lead ECG. Because there were two different hospital 12‐lead ECG devices in use during the study, we report results both by device and combined. Scatter plots were generated to evaluate the relationship between the measurement methods (bedside monitor versus standard 12‐lead ECG). In addition, Bland–Altman analyses were used to evaluate the agreement between the measurement methods (bedside monitor versus standard 12‐lead ECG). (Altman and Bland 1983) This approach plots the mean differences for QT/QTc between the two methods against the average of the two measurements. A mean difference in zero or close to zero indicates strong agreement. Unlike scatter plots, the Bland–Altman test can uncover measurement bias if one of the two methods is systematically inaccurate at capturing values at either end of the range of values for QT/QTc measurements. The Bland–Altman analysis identifies the estimated difference between the two measurements with 95% limits of agreement (LOA) around the estimate (mean difference in ±1.96 SD) and was conducted in R v4.0.0 using the BlandAltmanLeh package v0.3.1. (Altman and Bland 1983; Lehnert 2015; R Core Team 2020) The mean difference and confidence intervals were determined by a linear mixed model using lme4 v1.1.27.1 (R Core Team 2020). *p*‐Values < 0.05 were considered statistically significant.

## 3 Results

On the 5 days of data collection, we screened 120 patients for inclusion in the study. However, only 60 (50%) had a standard 12‐lead ECG recorded available for comparison. In many of these cases, a standard 12‐lead ECG had been obtained in the emergency department, or in a non‐ICU setting (i.e., step‐down, medical surgical unit), prior to ICU admission. As shown in Table 3, of the 60 patients, 33 (55%) were male, 27 (45%) were female, and the mean age was 58 (±18 years). The ICU type was cardiac (38%); medical–surgical (33%); and neurological (28%). The mean time difference between measurements (bedside monitor versus 12‐lead) was 7 min 51 s (±38 min). There were two patients with ECG time differences > 30 min (one 4 h and one 3 h), but as shown in Table 3 the QT/QTc measurements were similar. The overall mean QT in the sample was 377 ± 63 ms, and the overall mean QTc was 455 ± 50 ms.

**TABLE 3:** Sample characteristics among 60 intensive care unit patients.

Characteristic *n* = 60	*n* (%)			
Intensive care unit type				
Cardiac (28 beds)	23 (38)			
Medical–surgical (32 beds)	20 (33)			
Neurological (29 beds)	17 (28)			
Age in years (mean ± SD)	58 ± 18			
Sex				
Male	33 (55)			
Female	27 (45)			
Time difference (mean ± standard deviation) between bedside ECG and standard 12‐lead	7 min 51 s (±38 min) Two patients with > 30 min time difference—see QT/QTc measurements below			

Overall mean ± standard deviation QT/QTc in the sample in milliseconds (ms)				
	Bedside monitor (6 ECG leads)	Standard 12‐lead		
QT	380 ± 62 ms	377 ± 63 ms		
QTc	459 ± 40 ms	455 ± 50 ms		

Two patients with > 30 min time difference between ECG recordings				
	Bedside	Standard	Bedside	Standard
	QT	QT	QTc	QTc
#1 (time difference 4 h)	392	362	519	503
#2 (time difference 3 h)	312	284	461	444

Abbreviations: ECG = electrocardiogram; min = minutes; sec = seconds.

### 3.1 QT and QTc Measurement Comparisons by Standard 12‐Lead ECG Device

**FIGURE 1:**
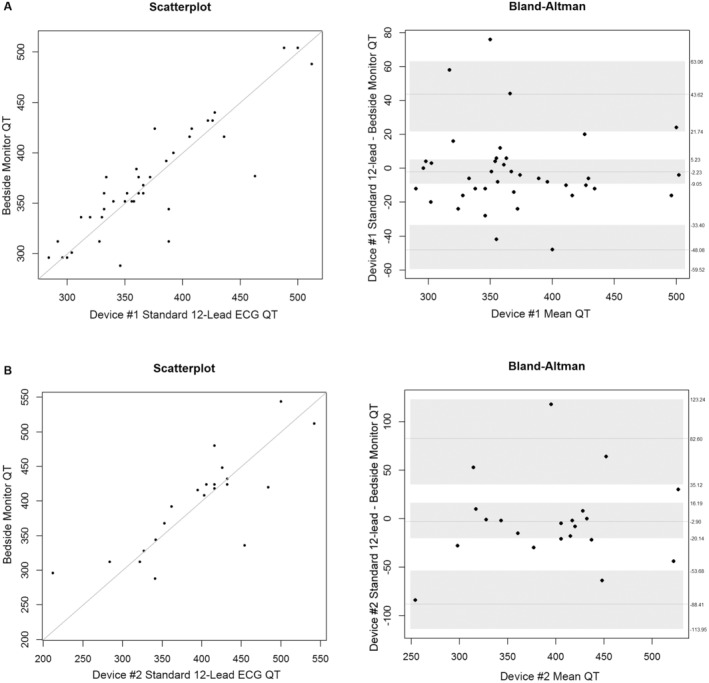
QT measurements using standard 12‐lead ECG device #1 (A, *n* = 39 patients) and device #2 (B, *n* = 21 patients). Shown are scatterplots (left) and Bland–Altman plots (right) comparing computerized measurements from the bedside monitor (four lead) to the standard 12‐lead ECG for each device. The line in the middle of the Bland–Altman figure represents the mean difference, and the gray shading is the upper and lower limits for the 95% confidence interval (CI) around the mean difference. The lighter dashed lines above and below the mean difference are the upper and lower limits where 95% of the data lie.

During the study, our hospital transitioned to a different standard 12‐lead ECG device. Therefore, we examined QT/QTc differences by device. Device #1: As shown in Table 4 and Figures 1 (QT) and 2 (QTc), the mean bias difference for measurements between the bedside monitor (four leads) versus the standard 12‐lead was not statistically different. Device #2: As shown in Table 4 and Figures 1 (QT) and 2 (QTc), the mean bias difference for measurements between the bedside monitor (four leads) versus the standard 12‐lead was not statistically different.

**TABLE 4:** Bland–Altman analysis of QT and QTc measurement comparisons by standard 12‐lead ECG device used. The values shown are in milliseconds. The *p*‐value reports the test of the mean bias using a linear mixed model.

Comparison group	Bias (mean)	95% CI	95% LOA	*p*
			Lower, upper	
Device #1 (*n* = 39)				
QT				
Standard 12‐lead vs. bedside monitor	−2.23	5.23 to 9.05	−48.08 to 43.62	0.53
Standard 12‐lead vs. bedside monitor	−2.90	16.19 to 20.14	−88.41 to 82.60	0.74
Device #2 (same manufacturer as bedside monitor) *n* = 21				
QT				
Standard 12‐lead vs. bedside monitor	−3.51	4.64 to 11.26	54.94 to 47.92	0.28
QTc				
Standard 12‐lead vs. bedside monitor	−2.62	16.62 to 19.48	−87.12 to 81.88	0.74

Abbreviations: CI = confidence interval; LOA = limits of agreement.

**FIGURE 2:**
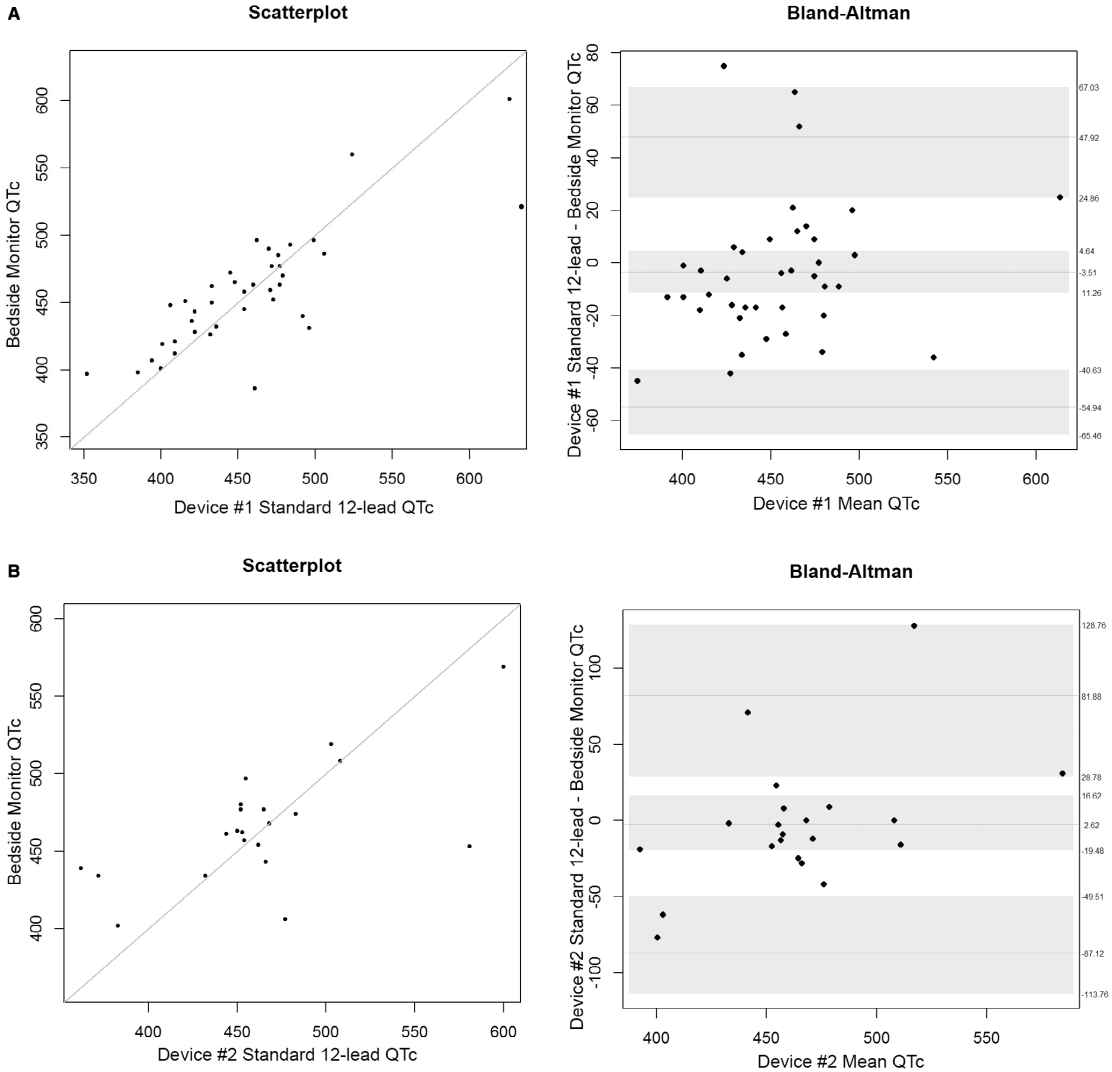
QTc measurements using standard 12‐lead ECG device #1 (A, *n* = 39 patients) and device #2 (B, *n* = 21 patients). Shown are scatterplots (left) and Bland–Altman plots (right) comparing computerized measurements from the bedside monitor (four lead) to the standard 12‐lead ECG for each device. The line in the middle of the Bland–Altman figure represents the mean difference, and the gray shading is the upper and lower limits for the 95% confidence interval (CI) around the mean difference. The lighter dashed lines above and below the mean difference are the upper and lower limits where 95% of the data lie.

### 3.2 Overall QT/QTc Measurement Comparisons—Bedside Monitor Versus Standard 12‐Lead

**FIGURE 3:**
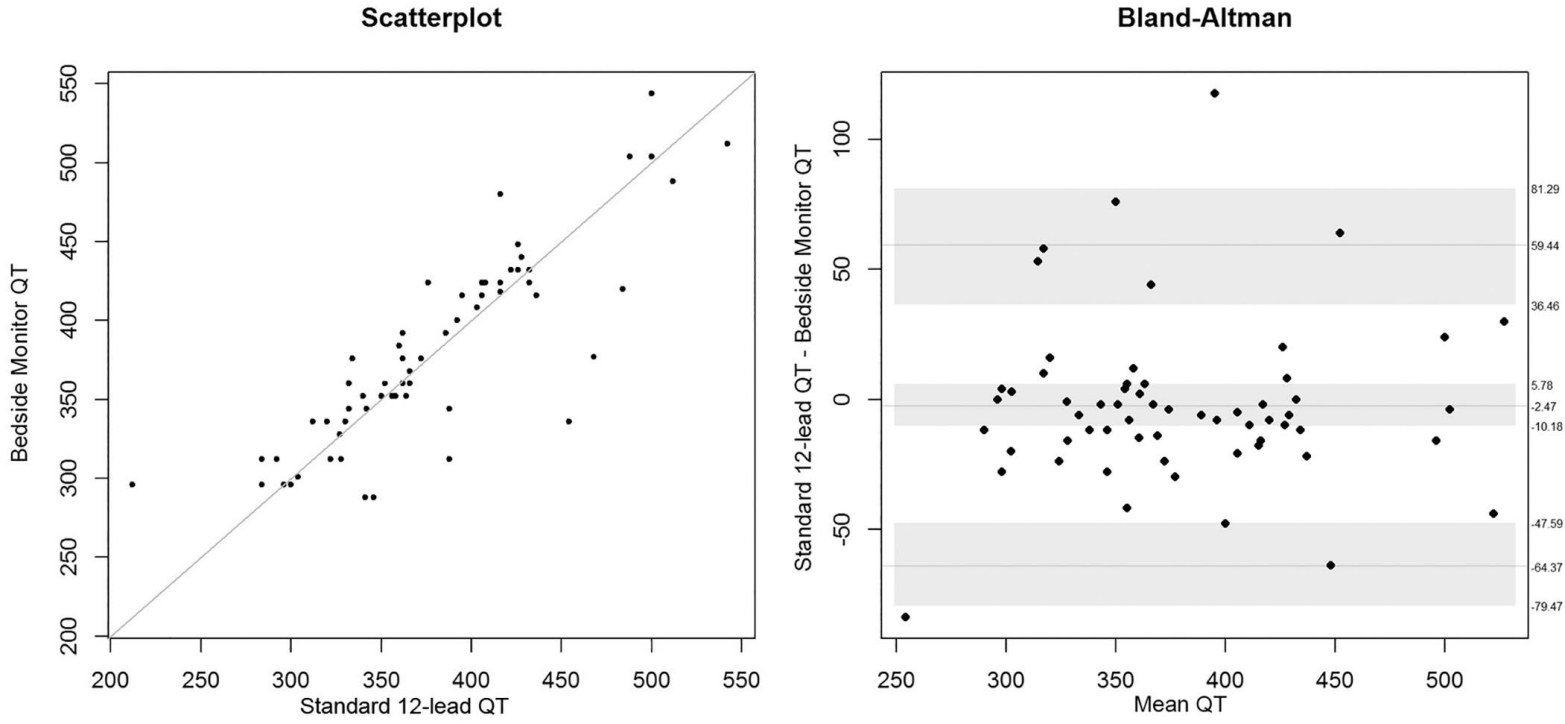
QT measurements in the overall sample (*n* = 60 patients), both device #1 and #2. Shown is a scatterplot (left) and Bland–Altman plot (right) comparing computerized measurements from the bedside monitor (four leads) to the standard 12‐lead ECG. The line in the middle of the Bland–Altman figure represents the mean difference, and the gray shading is the upper and lower limits for the 95% confidence interval (CI) around the mean difference. The lighter dashed lines above and below the mean difference are the upper and lower limits where 95% of the data lie.

**FIGURE 4:**
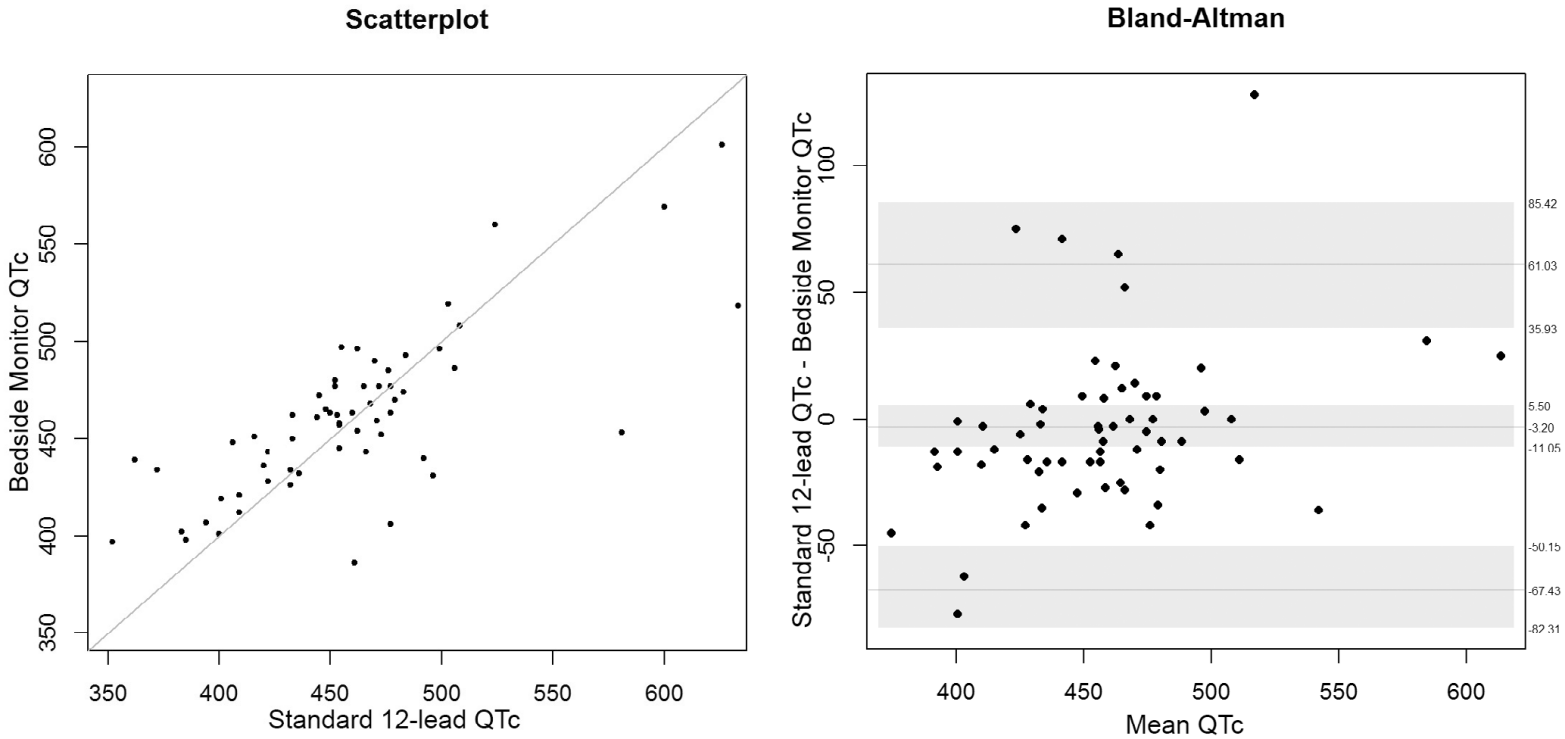
QTc measurements in the overall sample (*n* = 60 patients), both device #1 and #2. Shown is a scatterplot (left) and Bland–Altman plot (right) comparing computerized measurements from the bedside monitor (four lead) to the standard 12‐lead ECG. The line in the middle of the Bland–Altman figure represents the mean difference, and the gray shading is the upper and lower limits for the 95% confidence interval (CI) around the mean difference. The lighter dashed lines above and below the mean difference are the upper and lower limits where 95% of the data lie.

**FIGURE 5:**
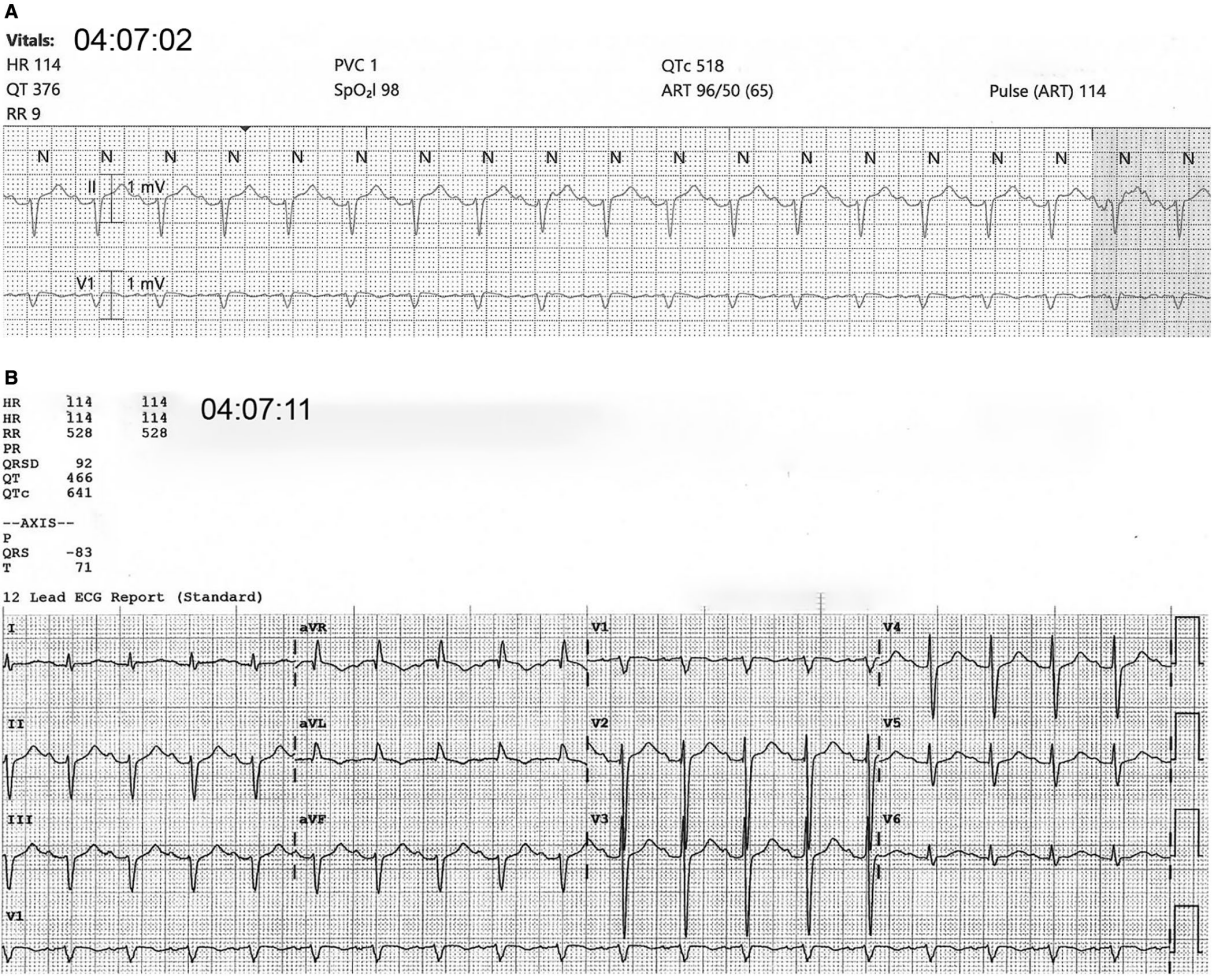
(A, B) Illustrates discordant computerized QT/QTc measurements with the longest QTc obtained in a 68‐year‐old male patient admitted to the cardiac intensive care unit. Note that the ECGs were obtained within 9 s of each other. The underlying rhythm is sinus tachycardia with a first degree atrioventricular block. Figure (A) is from the bedside ECG monitor in lead II (top tracing) and V1 (bottom tracing). The QT is 376 ms, and the QTc is 518 ms. Figure (B) is a standard 12‐lead ECG with a QT of 466 ms and QTc of 641 ms. The QT/QTc measurements from the standard 12‐lead ECG appear to include the P‐wave; thus, the measurement is inaccurate, whereas the bedside monitor measurements appear to correctly measure to the end of the T‐wave prior to the P‐wave and are more reliable.

Here, we report the overall QT and QTc analysis combining data from both device #1 and device #2. As shown in Table 5 and Figure 3, the mean bias difference for QT measurements between the bedside monitor (four leads) versus both standard 12‐lead devices was not statistically different (−2.47 ms; 95% CI + 5.78 to −10.18 ms; *p* = 0.53). As shown in Table 5 and Figure 4, the mean bias difference for QTc. Figure 5A,B, illustrates discordant QT/QTc measurements (bedside monitor versus standard 12‐lead) in a patient with the longest QTc.

**TABLE 5:** Bland–Altman analysis of QT and QTc measurement comparisons in the overall sample combining device #1 and #2. The values shown are in milliseconds. The *p*‐value reports the test of the mean bias using a linear mixed model.

Comparison group	Bias (mean)	95% CI	95% LOA	*p*
			Lower, upper	
QT				
Standard 12‐lead vs. bedside monitor	−2.47	5.78 to −10.18	−64.37 to 59.44	0.53
QTc				
Standard 12‐lead vs. bedside monitor	−3.20	5.50 to −11.05	−67.43 to 61.03	0.44

Abbreviations: CI = confidence interval; LOA = limits of agreement.

## 4 Discussion

In this study that included 60 ICU patient, we found good agreement between QT and QTc measurements when comparing those generated from the bedside ICU monitor, using only four ECG leads, to those obtained from a standard 12‐lead ECG. These data suggest that continuous and automatically generated QT/QTc's from bedside monitors are comparable to those of a standard 12‐lead ECG and may be a useful alternative. However, our sample was small and only included ICU patients, which limits the generalizability of our findings and is not sufficient for instituting a major practice change until a larger sample, including non‐ICU patients who are more mobile than ICU patients, are examined.

The mean QT bias of 2 ms reported in our study was less than that reported by Helfenbein et al. who found a mean bias difference in 8.1 + 40 ms (Altman and Bland 1983). In their study, 95 computerized QT intervals were compared to those measured by a cardiologists using a two lead ECG during a 15 min recording. In a separate study that examined the agreement between computerized bedside QT/QTc's to those measured by both bedside and expert nurses found that both nursing groups consistently measured longer QT/QTc's as compared to computerized measurements (Ho et al. 2023). However, bedside nurses measured shorter QT/QTc's than expert nurses, suggesting that measurement variability can occur between nurses (both bedside and experts) as compared to computer generated measurements. These findings are similar to a different study showing that there was higher variability between manual physician measurements as compared to computerized measurements (Janssen, Rijkenberg, and van der Voort 2016). An overall interpretation is that mixing computerized and nurse/physician measured QT/QTc's can vary; thus, in clinical practice a consistent measurement technique is important and is in line with current practice recommendations (Sandau et al. 2017). These above studies using human measurements support the use of computerized derived values because of the variability inherent in human measurements.

In our study, despite a mean time difference in 7 min when comparing bedside monitor derived measurements to a standard 12‐lead ECG, we did not find statistically different mean bias differences, which suggests that using these two methods interchangeably is likely to yield similar QT/QTc values. It is worth noting, that in our study there were two different standard 12‐lead ECG devices during the data collection period. This occurred because our hospital had purchased new devices from a different vendor. Despite two different standard 12‐lead ECG devices, we did not find statistical differences for QT of QTc measurement between standard 12‐lead ECG and those from the bedside monitor. Again, suggesting that computerized bedside monitor derived measurements are similar to standard 12‐lead ECG device measurements.

Mean bias differences for QTc were also not statistically different. Our results are similar to those of Janssen et al., who compared bedside monitor derived QTc's to a standard 12‐lead in 119 ICU patients. In their study, the mean bias was 7.8 ms, whereas in our study the mean bias difference was 3.20 ms, which does not appear to be clinically significant. In their study, patients with a QRS < 120 ms were examined, whereas in our study we did not account for QRS duration, which is a limitation of our study. However, their study included mostly cardiothoracic patients, whereas our study had an evenly distributed group of cardiac, medical/surgical, and neurological ICU patients.

### 4.1 Limitations

There are several limitations worth noting. First, our study included a small sample of 60 patients and only included ICU patients, which limits the generalizability of our findings. It is worth noting that we could have included as many as 120 patients on the 5 days we collected data. However, half of the patients we screened for inclusion did not have a standard 12‐lead ECG recorded during their ICU stay, which creates selection bias. Rather, the standard 12‐lead ECG had been obtained in the emergency department, and/or another hospital unit (step‐down, or medical surgical unit) prior to ICU admission. We were somewhat surprised by this finding since at one time it was common practice to record a daily standard 12‐lead ECG, and/or following a change in a patient's condition (i.e., acuity level, electrolyte disturbance, vital sign changes, arrhythmia, QT prolonging medication and/or symptoms), which is common in ICU patients. Whether this practice is similar at other hospitals is an important consideration and is another limitation of our study. Regardless of the rational for this practice, our data suggest that QT/QTc interval measurements are often not assessed using a standard 12‐lead ECG in a substantial number of ICU patients, which again may be hospital specific. However, this does imply that nurse measured (each shift) and those from the bedside monitor, if available, become an important assessment tool when a standard 12‐lead ECG has not been recorded. A future study where standard 12‐lead ECGs are obtained by research personnel that are compared to computerized QT/QTc's from the bedside monitor at the same time is needed.

One could also argue that a limitation was that we did not compare manual nurse/physician measurements to the bedside and standard 12‐lead ECG derived measurements. However, our group examined this in a different study and showed good agreement between nurse measured, both bedside and expert nurses, to computerized bedside ICU monitor derived QT/QTc's (Ho et al. 2023). This study builds on those findings by showing that bedside ICU monitor derived QT/QTc's, even when measured in only four leads, is in good agreement to those generated from a standard 12‐lead ECG. Another limitation was ECG time differences > 30 min in two patients, one 4 h and one 3 h. While the QT/QTc measurements were similar in these two patients, given the dynamic nature of QT/QTc values in ICU patients, using QT/QTc measurements in real‐world practice that are hours apart is problematic. Additionally, we did not account for QT/QTc interval changes based on the specific type of ICU patient, which can influence QT/QTc intervals. For example, cardiac patients often receive medications that prolong the QT/QTc and neurological patients can have ECG abnormalities that prolong the QT/QTc (e.g., increased intracranial pressure). This limitation should be addressed in a future study. Finally, we used two different standard 12‐lead ECG devices because our hospital introduced a new vendor's device during the study. However, we did not find differences when we compared each device, albeit in a small sample of ICU patients.

Given that as many as 70% of ICU patients have one or more American Heart Association indication for QT/QTc interval monitoring (Pickham et al. 2010), careful and consistent assessment for QT/QTc prolongation is clinically important. We found that standard 12‐lead ECGs were often not recorded in our ICU sample; thus, nurse measured and/or those from the bedside monitor are often relied upon for this assessment. Using computerized values could save time (both nurse and provider), reduce costs and identify QT/QTc prolongation earlier so that prompt treatment, if indicated, can be administered. However, as shown in our ECG example (Figure 5A,B), careful human oversight is required to ensure the accuracy of computerized QT/QTc measurements, especially in patients with a QT/QTc > 500 ms, which is considered prolonged in hospitalized patients (Drew et al. 2010; Sandau et al. 2017).

## 5 Conclusions

While this study examined a small number of ICU patients at a single‐center during only 5 days of data collection, our findings suggest that QT/QTc measurements generated from bedside monitors using only four ECG leads may be an acceptable alternative to obtaining a standard 12‐lead ECG. Using bedside monitor derived QT/QTc measurements may reduce disruptions in clinical care, lower costs and provide useful real‐time data that could identify QT/QTc prolongation earlier, which is important given the often subtle and dynamic nature of the QT/QTc interval. However, a future study in a larger sample that includes non‐ICU patients is needed prior to an overall practice change.

## Author Contributions

All authors listed made had substantial contributions to the conception and design of the research; the acquisition, analysis, and interpretation of data; participated in writing and reviewing the intellectual content; approved the final version to be published; are in agreement to be accountable for all aspects of the work in ensuring that questions related to the accuracy or integrity of any part of the work are appropriately investigated and resolved.

## Conflicts of Interest

The authors declare no conflicts of interest.

## Institutional Review Board Approval

IRB# 21–34,690.

## Data Availability

Data are available on request from the authors.
